# Isolation and Characterization of Polysaccharides from Oysters (*Crassostrea gigas*) with Anti-Tumor Activities Using an Aqueous Two-Phase System

**DOI:** 10.3390/md15110338

**Published:** 2017-11-01

**Authors:** Kit-Leong Cheong, Li-Xuan Xia, Yang Liu

**Affiliations:** Guangdong Provincial Key Laboratory of Marine Biotechnology, STU-UNIVPM Joint Algal Research Center, Department of Biology, College of Science, Shantou University, Shantou 515063, Guangdong, China; klcheong@stu.edu.cn (K.-L.C.); 141xxia@stu.edu.cn (L.-X.X.)

**Keywords:** *Crassostrea gigas*, polysaccharides, aqueous two-phase system, anti-tumor activity

## Abstract

In this study, a simple aqueous two-phase system (ATPS) was employed for concurrent purification of oyster polysaccharides. The chemical structure and anti-tumor activities of purified oyster polysaccharides (OP-1) were also investigated. Under optimal ATPS conditions, oyster polysaccharides can be partitioned in the bottom phase with 67.02% extraction efficiency. The molecular weight of OP-1 was determined as 3480 Da. OP-1 is a (1→4)-α-d-glucosyl backbone and branching points located at *O*-3 of glucose with a terminal-d-Glc*p*. The anti-tumor activity assay showed that OP-1 exhibited good activities, including promotion of splenocyte proliferation, IL-2 release, and inhibition of HepG2 cell proliferation. Additionally, OP-1 had no in vivo toxicity. This finding suggests that ATPS is a much simpler and greener system, and it opens up new possibilities in the large-scale separation of active polysaccharides from oysters. OP-1 could be used by the health food and pharmaceutical therapies as potential anti-cancer adjuvants.

## 1. Introduction

Pacific oysters (*Crassostrea gigas*) are the largest molluscan group cultured in China. They grow fast and spread quickly due to their good tolerance of environmental alterations [1]; this feature has helped them become an abundant ocean-derived resource. Pacific oysters are referred to as “the milk of the sea” because they are an excellent source of high-quality nutrition, which include polysaccharides, protein, peptides, lipids, phenolic compounds, and minerals [2,3]. In addition to their nutritional value, oysters could be utilized as potential functional food exhibiting beneficial health effects. Oysters are an adequate resource with high functional value; therefore, they have much potential in the functional food industry.

Functional studies have been conducted on oyster polysaccharides, including the potential interleukin-2 (IL-2) effects on peripheral blood mononuclear cells [4], modulation of antigen-induced splenocyte proliferation and T-cell cytokine expression [5], and enhancement of the phenoloxidase activity of hemocyte suspensions [6]. Recently, some bioactive polysaccharides have been isolated from oysters and have shown hepatoprotective [7], anti-hypertensive [8], and other anti-oxidant activities [9]. Usually, polysaccharides’ biological activities are closely correlated to their molecular mass, chemical structure, monosaccharide composition, and linkage type [10,11]. Therefore, a better understanding of oyster polysaccharides’ chemical structures and physical properties is essential for the successful interpretation of the correlation between the structure and bioactivity of these molecules and would also be beneficial for the future application of these types of polysaccharides in functional food areas.

Polysaccharides are a type of biomacromolecule that have wide applications in functional food, pharmaceutical, and industrial research fields. The method by which pure polysaccharides are effectively obtained is the basis of studies on this subject. The traditional methods for polysaccharide purification include ethanol precipitation, de-proteinization by the Sevag method, and ion-exchange and size exclusion chromatography [12,13]. However, these conventional methods are time-consuming, have laborious procedures and high operating costs, and involve organic solvents. An aqueous two-phase extraction system (ATPS) is a liquid–liquid separation technique based on the formation of two immiscible water-rich phases above certain critical concentrations of two mutually incompatible solutes [14]. ATPS has been successfully used to separate and purify flavonoids [15], small organic molecules [16], DNA [17], proteins [18,19], and polysaccharides [20] from fruits, plants, and herbs. APTSs are environmentally friendly, low-cost, easy to operate, and high in efficiency, and they have been used to separate and purify bioactive polysaccharides on a large scale in a single step. The most commonly explored type of ATPS in recent years for separation of polysaccharide/proteins is the polymer–salt system. High-molecular-weight hyaluronic acid was purified with a polyethylene glycol/potassium phosphate system, and nearly all of the hyaluronic acid in the salt-rich bottom phase and its purity reached almost 100% [21]. Xing and Li purified the aloe from bovine serum albumin with a PEG6000/(NH_4_)_2_SO_4_ system, and partitioned the aloe polysaccharides in the bottom phase. This phase separation and partitioning behaviour of aloe polysaccharides may be influenced by NaCl, pH, and Gu(SCN)_2_ [22]. A polymer-salt system consisting of ethylene-oxide-β-propylene-oxide-β-ethylene-oxide and NaH_2_PO_4_ was used to partition the *Lycium barbarum* extract. The *L. barbarum* polysaccharides were partitioned to the bottom phase with a high recovery ratio and purity [23].

In this study, a simple ATPS was employed to separate polysaccharides and proteins produced by Pacific oysters. The partitioning efficiency of polysaccharides and proteins and the influences of different processing parameters (such as composition and molecular mass of PEG) were analyzed in detail. The chemical structures and biological activities of the purified polysaccharides were also analyzed in detail. The results could be helpful in further understanding the structure–function relationship of these polysaccharides, and could pave the way for the potential use of oyster polysaccharides as functional foods or therapeutic agents.

## 2. Results and Discussion

### 2.1. Selection of the Optimal Aqueous Two-Phase System

To determine the optimal ATPS, the partitioning of oyster polysaccharides and proteins in ATPS at room temperature was investigated. The test systems are shown in Table 1. PEG/ammonium sulfate with different compositions and constant volume ratios were used. It can be seen that oyster polysaccharides were enriched in the bottom phase. A high polysaccharide extraction efficiency (about 71.84–89.42%) was found in the bottom phase of the PEG-1000/ammonium sulfate system. At the same time, the protein was also found in the bottom phase with an extraction efficiency of 64.43–83.38%. In the PEG-4000/ammonium sulfate system, the tie line length (TLL) increment caused a shift in the protein from the bottom to the top phase. The PEG-4000/ammonium sulfate system (2.2102 g:0.1319 g, *w*/*w*) with a TLL of 45 showed a relatively low protein extraction efficiency (about 15.59%) with a high polysaccharide concentration, and it showed that a large portion of protein was enriched in the top phase after the phase separation process. However, it may lose about 30.09% of polysaccharides in the top phase.

**Figure 1:**
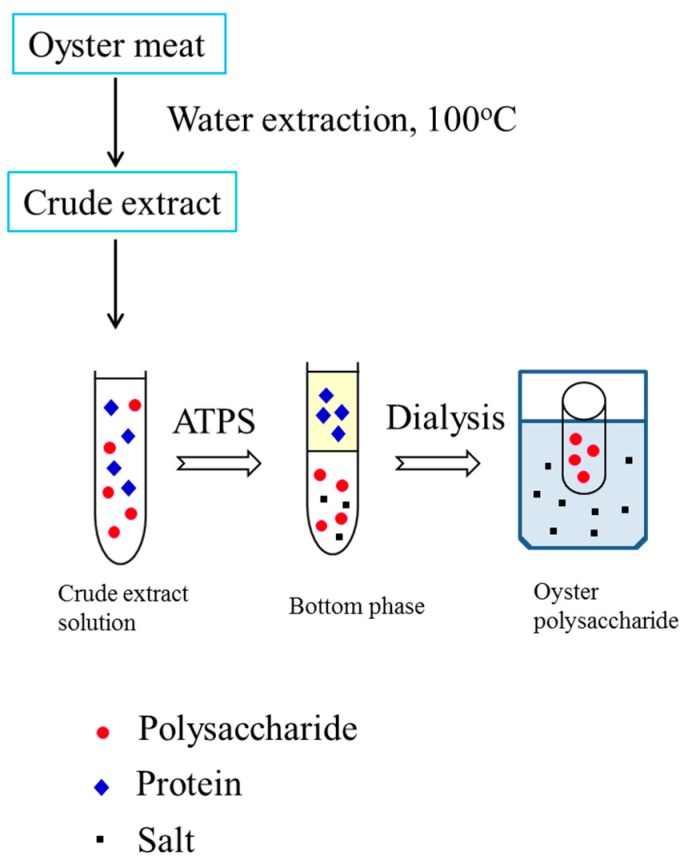
Flow chart of extraction, separation, and purification of polysaccharides from oysters using an aqueous two-phase system.

The ethanol/ammonium sulfate system with different compositions was further investigated. It can be seen that the ethanol/ammonium sulfate system had lower protein recovery in the bottom phase when compared with the PEG/ammonium sulfate system (Table 1) because more protein was in the middle phase. Accordingly, with an increase in TLL, the recovery of polysaccharides in the bottom phase decreased because the strong salting-out effect led to an increase in salt concentration. The increase in salt concentration led to less available free water to dissolve the oyster polysaccharides, resulting in decreased polysaccharide recovery. Therefore, 0.1770 g of ethanol and 0.2729 g of ammonium sulfate were selected for oyster polysaccharide partitioning. About 67.02% of polysaccharides were obtained in the bottom phase, and only 12.12% of the polysaccharides were lost in the top phase. After phase separation, the bottom phase was collected and dialyzed against water for 24 h. The retentate in the dialysis bag (a molecular weight cut-off of 500 Da) was concentrated in a vacuum evaporator and then lyophilized to dryness (Figure 1).

### 2.2. Purity and Homogeneity

The lyophilized purified polysaccharides were in the form of a white powder, named OP-1. The weight-average molecular weight (*M*_w_) and purity were analyzed by high-performance gel-permeation chromatography (HPPGC). A homogeneous peak appeared in the HPGPC elution profile of OP-1, with an *M*_w_ based on a column calibration of 3480 Da.

### 2.3. Chemical Structures of the Purified Polysaccharides

The monosaccharide composition of OP-1 is only glucose, as detected through the high performance liquid chromatography (HPLC) coupled with an evaporative light scattering detector (ELSD) of the OP-1 hydrolysate. The glycosyl-linkage of OP-1 was identified through methylation analysis. The GC-MS of the alditol acetates from methylated OP-1 revealed the presence of 1,5-di-*O*-acetyl-2,3,4,6-tetra-*O*-methyl-d-glucitol, 1,4,5-tri-*O*-acetyl-2,3,6-tri-*O*-methyl-d-glucitol, and 1,3,4,5-tetra-*O*-acetyl-2,6-di-*O*-methyl-d-glucitol derivatives at a molar ratio of 1.3:9.5:1. These results indicated that OP-1 exhibited an (1→4)-linked backbone with branching points located at *O*-3 of Glc*p* with terminal-d-Glc*p* as a side chain. Meanwhile, the degree of branching was calculated as 24%, according to the Tao et al. report [24]: DB=(NT+NB)(NT+NB+NL), where *N_T_*, *N_B_*, and *N_L_* are the total numbers of the terminal residues, branched residues, and linear residues, respectively.

**Figure 2:**
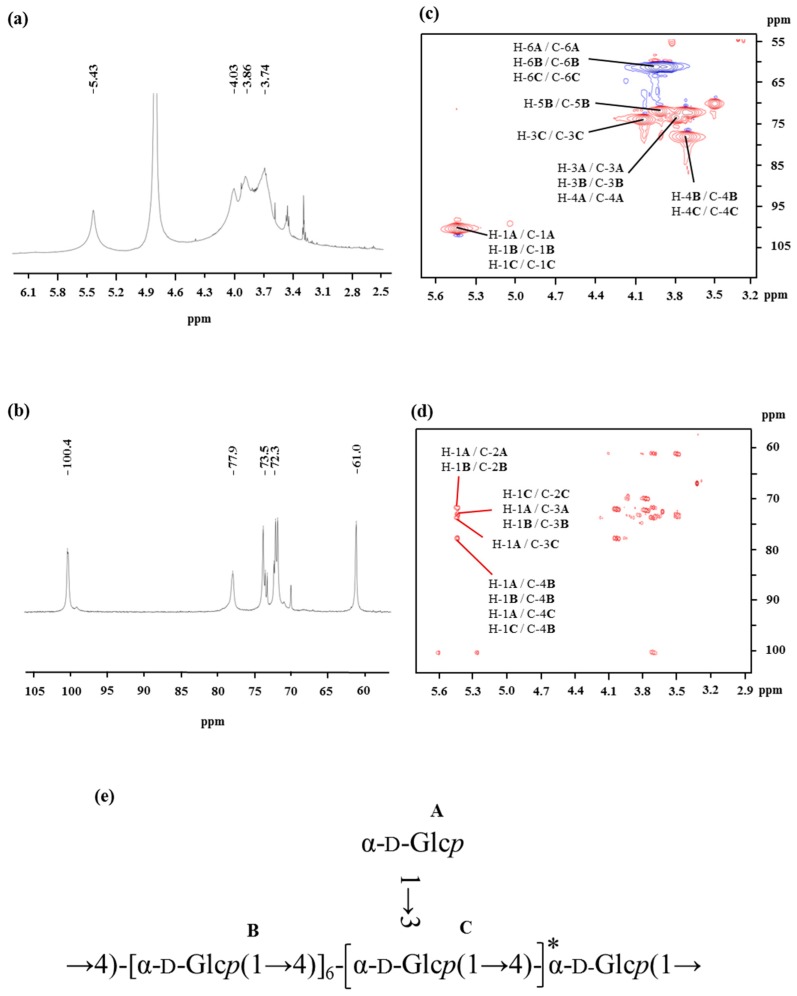
^1^H NMR (**a**), ^13^C NMR (**b**), HSQC (**c**), and HMBC (**d**) spectra of the OP-1 from oysters recorded in D_2_O at 27 °C. (**e**) The predicted structure of OP-1 isolated from the oyster.

The spectra of ^1^H NMR and ^13^C NMR of OP-1 are shown in Figure 2. The values of the chemical shifts found in the NMR spectrum of OP-1 are summarized in Table 2. The anomeric protons of Residues **A**, **B**, and **C** had chemical shifts larger than δ 5.00, suggesting that these residues were α-linked. The presence of residues of α-linked residues was identical to that found via FTIR. The signal around δ 5.43 overlapped with broad peaks, suggesting that there were some branches in α-(1→4)-linked residues [25]. The up-field signals between δ 3.9 and δ 3.4 were attributed to the proton resonances of H-2 to H-6. The signals in the ^13^C NMR spectra indicated that all of the residues (**A**, **B**, and **C**) of OP-1 share an α anomeric configuration based on the fact that the chemical shifts of glucose is δ 100.0 [26]. A signal observed at δ 77.6 ppm was assigned to the resonance of C-4 of 4-linked-α-Glc.

The HSQC (Figure 2c) and HMBC (Figure 2d) spectra of OP-1 provided anomeric intra-residue and inter-residue correlations, respectively. The HSQC technique provided correlations between the carbon and its attached protons (as listed in Table 2). The correlation signals can be employed as the starting point to completely assign all ^1^H and ^13^C signals of OP-1. Based on the HMBC interpretation, the chemical shifts of C-3, C-4, C-5, and C-6 of α-(1→4)-Glc*p* were assigned as δ 73.6, δ 78.0, δ 71.8, and δ 61.0, respectively. A down-field shift of signal of C-6 (*δ* 61.0), combined with the cross peak of *δ* 3.69 (H-4) with *δ* 78.0 in the HSQC spectrum, may indicate the presence of the α-Glc*p*-(1→ residues linked at the *O*-4 position. More structural information of OP-1 and the assignments of NMR resonances were recorded on the basis of analysis of HSQC and HMBC spectra. The inter-residue HMBC correlations from H-1 of residue **A** to C-4 of residue **B** (Figure 2d) indicated that the backbone of -[α-Glc*p*-(1→4)]- terminal linked with α-Glc*p*-(1→ residues. The linkage between Residues **B** and **C** was supported by the HMBC correlation of H-1 of Residue **C** with C-4 of Residue **B** (Figure 2d). Similarly, the cross-peak of H-1 (**A**) to C-3 (**C**) at δ 5.43/77.9, indicated that Residue **A** was substituted at the *O*-3 position of Residue **C** by side chains.

The NMR technique is a powerful tool for structural elucidation. However, it is limited and difficult to obtain the complete structure of polysaccharides by the interpretation of NMR spectra, for the NMR resonances of polysaccharides always overlap. Therefore, the proposed structure of OP-1 is mainly based on the data obtained from methylation and then further confirmed by NMR data. After comprehensive composition analysis, methylation analysis, and NMR experiments, it can be concluded that the repeating unit of OP-1 has a backbone of (1→4)-α-d-glucosyl residues, and has α-d-glucosyl residues on the terminal of the side chains at the *O*-3 position. The predicted structure of OP-1 is presented in Figure 2e.

More recently, the α-glucan from aquatic species showed interesting pharmacological activities has been attracting the attention of scientists seeking to explore new potent biological activities [27]. A water-soluble glucan isolated from *Cyclina sinensis* showed significant scavenging activities on superoxide radicals and an inhibitory effect in vitro on human gastric cancer cells [28]. A water-soluble α-glucan was isolated from foot muscle of *Bellamya purificata* by Zhang et al. [29], and significant anti-inflammatory activity was found. This glucan has a backbone of α-(1→4)-d-glucopyranosyl and branches at *O*-6 of α-(1→6)-d-glucopyranosyl residues [29]. Altogether, these data point to the idea that the α-glucan from mollusks contributes to the diversion of the immune host response.

### 2.4. Cell Proliferation and IL-2 Production

**Figure 3:**
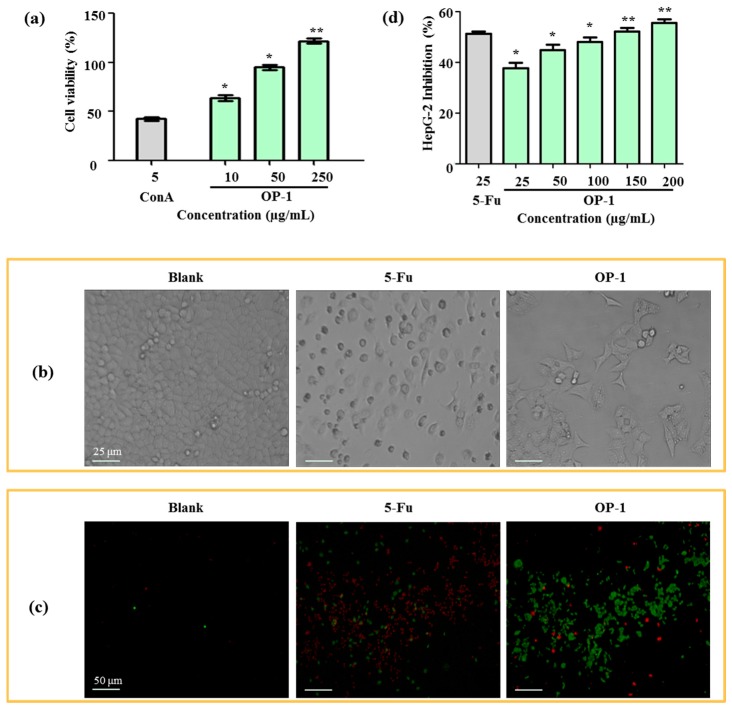
(**a**) Effects of OP-1 on the viability of spleen lymphocyte. (**b**) Morphology of HepG2 cells under inverted microscope. (**c**) Effects of OP-1 on phagocytosis of HepG2 cells in vivo. Fluorescence microscopic image of HepG2 cells were incubated with FITC-labeled annexin V and PI. (**d**) Inhibitory effect of OP-1 on HepG2 cells. * *p* < 0.05; ** *p* < 0.01 vs. control.

As the first step toward understanding the anti-tumor activity of OP-1, the effects of OP-1 on lymphocyte proliferation were investigated. As shown in Figure 3a, OP-1 was found to cause a dose-dependent increase at concentrations from 10 to 250 µg/mL in spleen lymphocyte cell proliferation. Cell proliferation was increased 64.44% when OP-1 at the concentration of 10 µg/mL, and ConA at the concentration of 5 µg/mL. The immune system plays a significant role in anti-tumor activity. Numerous reports have suggested that anti-tumor activity of the polysaccharides is mediated through a thymus-dependent immune mechanism [30]. Lymphocyte proliferation of splenocytes is a direct indicator of cellular immunity.

The activated immune cells also secrete some cytokines, such as IL-1, IL-2, IL-6, TNF-α, and IFN-γ. The effects of OP-1 treatment on the secretion of IL-2 are listed in Table 3. The results showed that OP-1 could increase lymphocyte secretion of IL-2. A similar result was observed in a study by Cheng et al. [5], in which it was demonstrated that lymphocytes exposed to oyster polysaccharides significantly increased the production of IL-4 and IFN-γ. Thus, OP-1 may have an indirect role in anti-tumor activity through cell proliferation promotion and the release of the lymphocyte-produced effector molecule, IL-2.

### 2.5. Cytotoxic Activity on HepG2 and Madin–Daby Canine Kidney (MDCK) Cell

After 24 h treatment with OP-1, human liver cancer HepG2 cells were examined by an inverted microscope. In Figure 3b, the number of cells in the OP-1 group obviously decreased when compared with the blank group (Figure 3b). OP-1 was shown to be dose-dependent on the inhibition of HepG2 cell proliferation (Table 4). At concentrations of 100 μg/mL and 200 μg/mL, the inhibitory ratios were 47.23% and 52.94%, respectively. In contrast to 5-fluorouracil (5-Fu), OP-1 had less anti-tumor activity on HepG2 cells (50.72% and 39.70%, respectively) when the same dose of OP-1 (25 μg/mL) was used.

The anti-tumor activity was further confirmed by a HepG2 phagocytic assay. Fluorescein isothiocyanate-conjugated (FITC)-labeled annexin V and propidium iodide (PI) uptake in HepG2 cells were compared between the positive control and the blank groups. HepG2 cells displayed phosphatidylserine on their outer cell membranes and were readily stained with annexin V in the early stages of apotosis. At later stages of apoptosis, PI moved across the cell membrane and bound to cellular DNA. Apoptotic cells could then be detected after they were double-stained with annexin V and PI. The results showed that OP-1 (15 µg/mL) could obviously enhance phagocytic activities, which displayed a higher FITC-fluorescence intensity when compared with the untreated control groups (Figure 3c,d).

Cytotoxicity assays for anti-tumor agents are important chemotherapeutic parameters. Therefore, cytotoxic effects were also determined in Madin–Daby canine kidney MDCK cells. OP-1 at doses of 25–250 μg/mL did not show cytotoxicity, while 5-Fu had a cytotoxic effect on MDCK cells (Table 4). The results suggest that OP-1 had no toxic effects at any of the tested concentrations.

Taking into account the increasing incidence of cancer in various organs, effective agents or compounds are urgently needed to control this problem. Most of the polysaccharides are considered to have a wide variety of biological activities. A brief review of recent advances in applications of polysaccharides derived from marine origin for pharmacological activities has been reported [31]. Over the last few decades, the functional food and pharmaceutical industries have shown a strong interest in polysaccharides derived from marine sources in general. The main reason for this increased interest is that polysaccharides are non-toxic, natural, less expensive, and abundantly available in nature. Therefore, oyster polysaccharides hold great promise as a potential source of new therapeutic agents.

Based on the above results describing its biological activities, OP-1 had significant anti-tumor activities against liver cancer and HepG2 cells and had no direct toxicity to the normal cells.

## 3. Materials and Methods

### 3.1. Materials

Acetic anhydride was purchased from Sigma-Aldrich (St. Louis, MO, USA). Dimethyl sulfoxide (DMSO; 99.9%) was purchased from Alfa Aesar (Ward Hill, MA, USA). Methyl iodide was purchased from VWR International (Lutterworth, UK). Other reagents were analytical grade.

### 3.2. Extraction of Polysaccharides from Oyster

Oyster meat was stripped from the shells, and then homogenized in a blender. The homogeneous mixture was extracted with water at 90 °C for 3 h. After cooling to room temperature, the extract was centrifuged at 4000× *g* for 15 min and the supernatant was collected. Total sugar content was determined by the phenol-sulfuric acid assay with glucose as the standard [32]. The protein concentration of each sample was determined by a BCA protein assay kit (Thermo Fisher Scientific Inc., Rockford, IL, USA).

### 3.3. Preparation of Aqueous Two-Phase Systems

Aqueous PEG/salt systems were prepared by weighing the appropriated amounts of PEG stock solution, ammonium sulfate stock solution, oyster extract, and water, to a final weight of 8 g, in order to achieve the desired composition. Ethanol/salt systems were prepared by weighing the appropriated amounts of ethanol, ammonium sulfate stock solution, oyster extract, and water to a final weight of 8 g. The two phases were mingled thoroughly and stood for 10 min, and the two phases were then completely separated. The partitioning experiments were carried out at room temperature.

The TLL was determined by the square root of the sum of the squares of the difference between the top and bottom phases. The volume ratio was obtained dividing the volume of top phase by the volume of the bottom phase. The extraction efficiency (*E*, %) was the concentration of the polysaccharides or proteins partitioned in the bottom phase to the amount of the sample added. The extraction efficiency was calculated with the following equation:

$$E\;\left(\%\right)=\frac{C_{t}\times V_{t}}{m_{o}}\times 100\%$$

where *C_t_* is the concentration of the upper phase, *V_t_* is the volume of the upper phase, and *m_o_* is the amount of the sample added.

### 3.4. Chemical Characterization

#### 3.4.1. Monosaccharide Composition Analysis

Polysaccharides (5 mg) were treated with 1 mol/L H_2_SO_4_ (2 mL) at 100 °C for 4 h. After hydrolysis, the mixture was neutralized with Ba(OH)_2_. The hydrolysate was detected by Agilent 1200 HPLC system (Agilent, Santa Clara, CA, USA) equipped with an ESLD (Alltech, Deerfield, IL, USA). Chromatographic separation was performed at 25 °C using a column of Prevail Carbohydrate ES column (250 mm × 4.6 mm, 5 μm, Alltech). The mobile phase consisted of acetonitrile and water (*v*/*v*, 75:25) at a flow rate of 1.0 mL/min. The drift tube temperature for ELSD was set at 83 °C, and the nitrogen flow rate was 2.1 L/min. The injection volume was 5 μL.

#### 3.4.2. Methylation Analysis

Purified polysaccharides were methylated according to a previous report [33]. The polysaccharides (5 mg) were dissolved in DMSO (5 mL); subsequently, 20 mg of NaOH and 150 μL of CH_3_I were added. After reaction for 4 h in the dark, water and dichloromethane were then added. The dichloromethane layer was dried under vacuum at 50 °C. Subsequently, the residue was hydrolyzed with TFA for 6 h at 105 °C. The hydrolyzed product was also dried under vacuum at 50 °C, and the residue was subsequently reduced with NaBH_4_, neutralized with acetic acid, and acetylated with acetic anhydride to obtain a mixture of partially *O*-methylated alditol acetates. Qualitative and quantitative analyses were conducted using GC-MS.

#### 3.4.3. ^1^H and ^13^C NMR Test

Purified polysaccharides (25 mg) were dissolved in 1 mL of D_2_O at room temperature. NMR spectra were recorded on a Bruker Avance 400 spectrometer (Bruker BioSpin, Billerica, MA, USA) at 400 MHz for ^1^H and 100 MHz for ^13^C.

### 3.5. Anti-Tumor Activities of Oyster Polysaccharide

#### 3.5.1. Preparation of Spleen Lymphocytes

The extirpated spleens were minced into small pieces with knife and suspended in an RPMI 1640 medium. Single-cell suspension was filtered by a sterile sieve mesh. The cells were supplemented with 6 mL of lysis buffer to remove the red blood cells, and then washed 3 times with cold phosphate-buffered saline. Subsequently, the cells were adjusted to a concentration of 1 × 10^6^ cells/mL in RPMI-1640 medium. The suspended cells were then collected.

#### 3.5.2. Cell Proliferation Assay

Cells (1 × 10^4^ cells/well) were treated with serial concentrations (10, 50, and 250 µg/mL) of OP-1 and 5.0 µg/mL ConA for 24 h, respectively. Cell culture RPMI-1640 medium was used as a blank group. Following incubation at 37 °C and 5% CO_2_, MTT (0.5 mg/mL) was added and co-incubated for an additional 4 h in the dark. After cultivation, the supernatants were removed, and the insoluble purple-colored formazan MTT was dissolved in sodium dodecyl sulfate lysis buffer (sodium dodecyl sulfate, isopropanol, and HCl). The absorbance values were recorded at 570 nm, and the cell viability was calculated as the percentage of the ratios of absorbance between the treated and blank groups.

#### 3.5.3. Measurement of IL-2

Cells (1 × 10^6^ cells/mL) were exposed to 250 µg/mL of OP-1 and 5.0 μg/mL ConA for 24 h at 37 °C and 5% CO_2_. After incubation, the cell supernatants were then collected via centrifugation at 1200× *g* for 5 min. The concentrations of IL-2 were assayed via an ELISA kit.

#### 3.5.4. HepG2 and MDCK Cells Proliferation Assay

HepG2 cells (4 × 10^4^ cells/mL) and MDCK cells (4 × 10^4^ cells/mL) were plated on 96-well plates, separately. After the cells were incubated for 24 h, the cells were supplemented with serial concentrations of OP-1 and 5-Fu, respectively. Cell culture DMEM medium was used as the control group. Following incubation at 37 °C and 5% CO_2_, MTT (0.5 mg/mL) was added and co-incubated for an additional 4 h in the dark. After cultivation, the supernatants were removed, and the residues were dissolved with DMSO. The absorbance values were recorded at 570 nm, and the percentage of cell inhibition (%) was calculated from the decrease in absorbance of treated samples as compared with the control cells, which is calculated using the following formula:

Cell inhibition (%) = (*A_c_* − *A_s_*)/*A_c_* × 100% where *A_c_* is the absorbance value of the control, and *A_s_* is the absorbance value of the test compound.

#### 3.5.5. Phagocytic Assay

FITC-labeled annexin V was used for the phagocytic assay of HepG2. Cells (4 × 10^4^) were cultured in a 96-well plate with additional OP-1 and 5-Fu for 24 h, respectively. The cell culture medium was used as a blank group. Then, the cells were washed with PBS and then stained with 200 μL of labeling solution containing FITC-labeled annexin V and PI for 10 min at room temperature. An observation was made at a final magnification of 400× using a fluorescence microscope. The phagocytic ability expressed as a mean fluorescence intensity (MFI)-FITC between the cells in treatments group and those of the blank group.

### 3.6. Statistical Analysis

Data were expressed as mean ± standard errors based on at least three independent experiments for each sample. Statistical significance was calculated via one-way analysis of variance test followed by a least significant difference test. Values of *p* <0.05 were considered to be statistical significant.

## 4. Conclusions

A simple and efficient ATPS has been successfully applied to separate oyster polysaccharides and proteins. Under optimum ATPS conditions, polysaccharides were separated in the bottom phase, and the extraction efficiency was 67.02%. This ATPS is a simple, fast, and green separation system that can be used to isolate and purify oyster polysaccharides on a large scale. These polysaccharides (OP-1) consisted of glucose and had an *M*_w_ of 3480 Da. OP-1 consisted of a backbone of (1→4)-α-d-glucosyl with α-d-glucosyl residues on the terminal of the side chains at the *O*-3 position. Moreover, OP-1 can stimulate the phagocytic activity and IL-2 production. OP-1 showed significant anti-tumor activity against HepG2 cells in vitro and had no direct toxicity to normal cells. These results can guide the direction of future research toward novel functional food or therapeutic agents.

## Figures and Tables

**Table 1:** Effect of different compositions of PEG, ethanol and ammonium sulfate, tie line length, and volume ratio on oyster polysaccharide content in aqueous two-phase systems.

Composition (*w*/*w*)		Tie Line Length	Volume Ratio	Recovery of Polysaccharides (%)		Recovery of Protein (%)	
				Top Phase	Bottom Phase	Top Phase	Bottom Phase
PEG-1000	AS ^a^						
0.2270	0.1764	30	1.02 ± 0.01	5.71 ± 0.31	83.88 ± 0.19	14.68 ± 0.25	83.38 ± 0.31
0.2348	0.1796	35	1.06 ± 0.04	6.92 ± 0.17	73.25 ± 0.27	8.59 ± 0.07	77.76 ± 0.25
0.2432	0.1834	40	1.03 ± 0.01	8.16 ± 0.28	89.42 ± 0.33	11.83 ± 0.12	55.47 ± 0.33
0.2520	0.1876	45	1.04 ± 0.02	8.92 ± 0.10	71.84 ± 0.32	20.09 ± 0.12	72.23 ± 0.23
0.2613	0.1923	50	1.03 ± 0.02	8.61 ± 0.13	72.60 ± 0.21	18.67 ± 0.27	64.43 ± 0.29
PEG-2000	AS						
0.1524	0.1153	30	1.06 ± 0.03	8.79 ± 0.22	69.61 ± 0.17	5.71 ± 0.22	83.88 ± 0.45
0.1697	0.1228	35	1.04 ± 0.04	6.25 ± 0.13	49.08 ± 0.23	6.92 ± 0.25	73.25 ± 0.33
0.1870	0.1306	40	1.02 ± 0.01	5.93 ± 0.05	29.50 ± 0.39	8.16 ± 0.09	89.42 ± 0.37
0.2045	0.1389	45	1.03 ± 0.02	5.55 ± 0.09	37.84 ± 0.26	3.92 ± 0.01	81.84 ± 0.31
0.2221	0.1477	50	1.02 ± 0.02	9.67 ± 0.14	36.02 ± 0.31	2.61 ± 0.01	72.60 ± 0.47
PEG-4000	AS						
0.1721	0.1102	30	1.03 ± 0.02	14.68 ± 0.17	83.38 ± 0.39	12.74 ± 0.01	52.64 ± 0.31
0.1841	0.1167	35	1.06 ± 0.04	8.59 ± 0.16	77.76 ± 0.43	12.77 ± 0.05	67.35 ± 0.22
0.1966	0.1238	40	1.05 ± 0.04	18.20 ± 0.08	73.46 ± 0.32	8.24 ± 0.01	52.86 ± 0.30
0.2102	0.1319	45	1.02 ± 0.02	30.09 ± 0.23	62.23 ± 0.28	36.26 ± 0.13	15.59 ± 0.27
0.2238	0.1404	50	1.05 ± 0.02	38.67 ± 0.32	54.43 ± 0.19	35.62 ± 0.16	10.44 ± 0.04
Ethanol	AS						
0.1770	0.2729	35	1.05 ± 0.03	12.12 ± 0.11	67.02 ± 0.33	25.01 ± 0.11	18.13 ± 0.29
0.1844	0.2749	40	1.02 ± 0.01	13.47 ± 0.18	65.35 ± 0.21	23.92 ± 0.34	21.28 ± 0.17
0.1930	0.2776	45	1.04 ± 0.01	12.24 ± 0.09	52.86 ± 0.44	44.69 ± 0.31	21.91 ± 0.13
0.2030	0.2811	50	1.04 ± 0.02	26.26 ± 0.37	45.59 ± 0.23	45.13 ± 0.42	22.08 ± 0.22
0.2120	0.2845	55	1.03 ± 0.03	25.62 ± 0.27	37.44 ± 0.23	45.62 ± 0.37	26.05 ± 0.21

^a^ AS: ammonium sulfate.

**Table 2:** ^1^H NMR and ^13^C NMR chemical shifts (δ) of the OP-1 from the oyster recorded in D_2_O at 27 °C.

Residues	Chemical Shifts ^1^H/^13^C (ppm)						
	1	2	3	4	5	6(a)	6(b)
A α-Glc*p*-(1→	5.43	3.74	3.74	3.79	3.74	3.94	3.86
	100.4	71.7	73.5	73.2	72.4	61	
B α-(1→4)-Glc*p*	5.43	3.64	3.7	3.69	3.91	3.94	3.86
	100.4	71.9	73.6	78	71.8	61	
C α-(1→3,4)-Glc*p*	5.43	3.68	4.03	3.73	3.71	3.94	3.86
	100.4	72.3	73.8	77.9	72.1	61	

Underlined bold numbers represent glycosylation sites.

**Table 3:** Effect of oyster polysaccharides (OP-1) on IL-2 secretion via lymphocyte.

Sample	IL-2 (pg/mL)	SI (%)
Blank	167.09 ± 3.92	—
Con A	239.46 ± 3.28	43.31 ± 2.73
OP-1	439.80 ± 2.72 **	149.87 ± 0.18 **

Mean ± SD; *n* = 3. ** *p* < 0.01 vs. control.

**Table 4:** Cytotoxicity of OP-1 at different concentrations against human liver cancer HepG2 cells and Madin–Daby canine kidney MDCK cells in vitro.

Concentration	HepG2 Cells		MDCK Cells	
μg/mL	5-Fu	OP-1	5-Fu	OP-1
25	50.72 ± 0.71	39.70 ± 3.18	42.65 ± 1.31	−6.05 ± 0.96
50		46.20 ± 2.61		−4.50 ± 1.13
100		47.23 ± 2.03		−9.78 ± 4.00
150		52.24 ± 1.16		−14.01 ± 0.30 *
200		55.82 ± 2.03		−15.48 ± 6.65 **

Mean ± SD; *n* = 3. * *p* < 0.05; ** *p* < 0.01 vs. control.
